# Characterization of the Retinal Phenotype Using Multimodal Imaging in Novel Compound Heterozygote Variants of CYP2U1

**DOI:** 10.1016/j.xops.2024.100618

**Published:** 2024-09-11

**Authors:** Ferenc B. Sallo, Chantal Dysli, Franz Josef Holzer, Emmanuelle Ranza, Michel Guipponi, Stylianos E. Antonarakis, Francis L. Munier, Alan C. Bird, Daniel F. Schorderet, Beatrice Rossillion, Veronika Vaclavik

**Affiliations:** 1 Oculogenetic Unit, Jules Gonin Eye Hospital, University of Lausanne, Lausanne, Switzerland; 2 Department of Ophthalmology, Inselspital, University of Bern, Bern, Switzerland; 3 Department of Neurology, University Hospitals of Geneva, Geneva, Switzerland; 4 Department of Genetic Medicine and Development, University of Geneva, Geneva, Switzerland; 5 Department Medical Retina, Moorfields Eye Hospital NHS Foundation Trust, London, United Kingdom; 6 Institute for Research in Ophthalmology, Sion, Switzerland; 7 Department of Ophthalmology, University of Lausanne, Lausanne, Switzerland; 8 Faculty of Life Sciences, Ecole Polytechnique Federal de Lausanne, Lausanne, Switzerland; 9 Vision Rive Droite SA, Grand-Saconnex, Geneva, Switzerland; 10 Ophthalmology Department, Hôpital Cantonal de Fribourg, HFR, Fribourg, Switzerland

**Keywords:** *CYP2U1*, Hereditary Spastic Paraplegia type 56, MacTel, Maculopathy, Multimodal imaging

## Abstract

**Purpose:**

To report the retinal phenotype in 2 patients simulating type 2 macular telangiectasis with new variants in *CYP2U1* implicated in hereditary spastic paraplegia type 56 (HSP 56).

**Design:**

Cross sectional case series study.

**Participants:**

Five members of a non-consanguineous family (parents and 3 male children) were investigated.

**Methods:**

All family members underwent a full ophthalmic evaluation and multimodal retinal imaging. Two family members demonstrating retinal anomalies underwent additional OCT angiography, dual wavelength autofluorescence and fluorescence lifetime imaging ophthalmoscopy, kinetic perimetry, fundus-correlated microperimetry, electroretinography, and electro-oculography. Whole-exome sequencing was performed in all 5 family members.

**Main Outcome Measures:**

To characterize the retinal phenotype in affected patients with variants in *CYP2U1*, using multimodal imaging: dual-wavelength autofluorescence, fluorescence lifetime, OCT angiography.

**Results:**

The 2 siblings with compound heterozygous novel variants c.452C>T; p.(Pro151Leu), c.943C>T; p.(Gln315Ter) in *CYP2U1* demonstrated parafoveal loss of retinal transparency and hyperreflectivity to blue light, redistribution of macular pigment to the parafoveal edge, photoreceptor loss, and fluorescence lifetime imaging ophthalmoscopy anomalies: a pattern compatible with that seen in macular telangiectasia type 2 (MacTel). One had manifest neurological abnormalities since early childhood; the second had no neurological abnormalities. Each parent and the third sibling were heterozygous for 1 variant and were neurologically and ophthalmically normal.

**Conclusions:**

These *CYP2U1* variants are associated with a retinal phenotype very similar to that otherwise specific for MacTel, suggestive of possible links in the etiology and pathogenesis of these diseases.

**Financial Disclosure(s):**

The author(s) have no proprietary or commercial interest in any materials discussed in this article.

Hereditary spastic paraplegia (HSP), also known as Strümpell-Lorrain disease, is a neurological disease characterized by either an isolated pyramidal syndrome (pure form) or in association with other neurological or extra neurological signs (complex form).^1^ Pure HSP typically presents with progressive bilateral lower leg spasticity and weakness. Hereditary spastic paraplegias are among the most clinically and genetically heterogeneous group of inherited neurodegenerative disorders. Hereditary spastic paraplegias are transmitted by all Mendelian forms of inheritance, including a dominant transmission of de novo variants. More than 70 causative genes and/or loci have been identified so far.^2^

Hereditary spastic paraplegia 56 (SPG56 MIM 615030, also known as SPG49), is an autosomal recessive form of HSP because of biallelic pathogenic variants in *CYP2U1* (MIM # s610670) that may manifest as a pure or a complex form. *CYP2U1* encodes a member of the cytochrome P450 group of enzymes. This protein is involved in lipid metabolism and has also been implicated in mitochondrial function.^3^ The phenotypic spectrum of SPG56 has been expanded since the first description; to date, 32 affected individuals have been reported with pathogenic variants in *CYP2U1*.^4^, ^5^, ^6^, ^7^, ^8^, ^9^, ^10^, ^11^, ^12^, ^13^, ^14^ Additional clinical features may include cerebellar ataxia, dystonia, intellectual disability, cognitive delay, visual impairment, and subclinical peripheral neuropathy. Brain magnetic resonance imaging may (rarely) show a thin corpus callosum, calcification of basal ganglia, and delayed myelination.^5^^,^^6^^,^^12^^,^^13^ Although visual impairment is a frequent complication of HSPs,^15^ a pigmentary maculopathy has only been described in a few families^5^^,^^10^ and isolated cases.^7^^,^^16^

Macular telangiectasia type 2 (MacTel) is a progressive degenerative neuroglial-vascular macular disease that leads to loss of central vision, with a late onset of symptoms and a characteristic constellation of retinal signs.^17^^,^^18^ The condition has a strong genetic component, supported by extended families with multiple affected individuals, linkage, and genome-wide association studies; however, the inheritance pattern is not clear because of the variable penetrance and expressivity of the disease.^19^, ^20^, ^21^, ^22^

In this study, we aim to characterize the retinal phenotype of 2 male siblings with compound heterozygosity for 2 novel pathogenic and likely pathogenic *CYP2U1* variants, investigate the similarities of their retinal phenotype to that of MacTel, and discuss the potential implications.

## Methods

### Patient Selection and Clinical Evaluation

**Figure 1:**
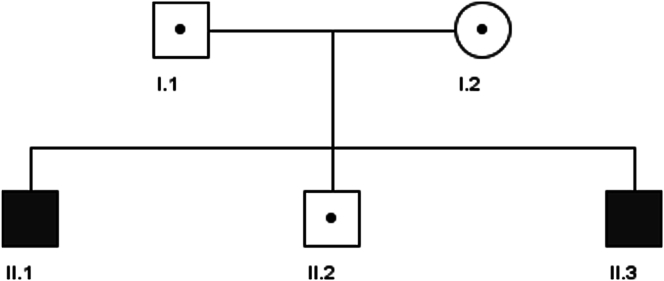
Pedigree of the family. Filled symbols represent siblings affected by maculopathy. Dots represent CYP2U1 carrier states.

The protocol of the study adhered to the tenets of the Declaration of Helsinki and was approved by the local Ethics Committee (CER-VD, Lausanne, Switzerland, Req-2019-00518). Written informed consent was obtained from all patients before inclusion in the study. Five members of a Swiss White, non-consanguineous family of Italian origin were examined (Fig 1). At the time of baseline ophthalmic imaging, the father (I.1) was aged 56, the mother (I.2) was 57, the eldest sibling (II.1) was 25 years old and, siblings II.2 and II.3 were 24 and 23 years old, respectively. All siblings were male. The eldest sibling (II.1) developed gait abnormalities at an early age and was diagnosed with Strümpell-Lorraine disease at an early age, prompting a genetic screening of the entire family.

### Whole Exome Sequencing

The whole exome sequence of Individual II.1 was performed using the following procedure: DNA was captured, and coding regions and splice sites were enriched using the Agilent SureSelect QXT Human All Exon V5 capture kit. Sequencing was performed on an Illumina instrument following the manufacturer’s protocol. Targeted bioinformatics analysis of a panel of 137 genes involved in spastic paraplegia (HSP_v1.00_137 genes_ER.txt_.HUGO) was done through locally developed pipelines, integrating BWA v0.7.10, Picard 1.80, GATK 3.50, and ANNOVAR vSept2015. We selected only the variants from the genes of interest, masking the rest of the data. The evaluation of variants used the following databases: dbSNPv142, ExAC 0.3, gnomAD 2.0, ClinVar 2016, HGMD 2016, LOVD, and the local database of variants. Prediction programs used were as follows: SIFT, Polyphen2, MutationTaster, dbscsnv11, and HSF v.3.0. Variants were classified according to the recommendations of the American College of Medical Genetics.^23^ Variables detected were validated, and familial segregation was performed by Sanger sequencing. The same variants were validated in patient II.3.

### Ophthalmic Phenotyping

All family members underwent a full ophthalmic evaluation, including Goldmann applanation tonometry, slit lamp examination, indirect ophthalmoscopy, and color fundus (CF) imaging, using a Topcon TRC50DX retinal camera (Topcon Co) and a Zeiss Clarus 500 Retinal Camera (Carl Zeiss Meditec AG). OCT, 488nm fundus autofluorescence (AF), blue light reflectance (BLR), imaging and infrared (IR) imaging were performed using Heidelberg Spectralis OCT + HRA devices (Heidelberg Engineering).

Two family members with detectable retinal anomalies underwent additional retinal imaging, including OCT angiography (OCTA) using an AngioVue device (Optovue Inc). Dual wavelength AF (DWAF) imaging for measurement of macular pigment optical density (excitation wavelengths: 488 nm and 518 nm) and Fluorescence Lifetime Imaging Ophthalmoscopy (fluorescence lifetime imaging ophthalmoscopy, excitation wavelength: 473 nm, detection range: 498-560 nm) were performed using custom Heidelberg Spectralis devices (Heidelberg Engineering). Fluorescence Lifetime Imaging Ophthalmoscopy is used to detect the decay time of natural retinal fluorophores after excitation by a blue laser light.^24^^,^^25^ The combination of autofluorescence intensity and fluorescence lifetime distribution provides additional information, especially from weak retinal fluorophores.

### Functional Testing

Functional testing included best corrected visual acuity (BCVA) measurements to the ETDRS standard at 4 meters, kinetic perimetry (Octopus 900, Haag-Streit AG Diagnostics), and automated, fundus-correlated microperimetry using a Centervue macular integrity assessment system device (Icare Finland Oy), and a 95-point test pattern developed for the MacTel CNTF phase II study.^26^ “En face” OCT images of the ellipsoid zone (EZ) and microperimetric retinal sensitivity threshold data were aligned, thresholds measured within EZ breaks were compared with those outside lesions.^27^ Results are reported in decibels. Fixation stability was expressed as the bivariate contour ellipse area, a standardized measure that provides a means for quantification and comparison of fixation stability.^28^

Full-field electroretinograms (ERGs) were performed after pupil dilation using 1% tropicamide and 2.5% phenylephrine hydrochloride, according to International Society for Clinical Electrophysiology of Vision standards, using an Espion Visual Electrophysiology System (Diagnosys Vision Ltd). The protocol included rod-specific and standard bright flash ERG, both recorded after a minimum of 20 min of dark adaptation. Photopic 30Hz flicker cone and transient cone ERGs were recorded after 10 minutes of light adaptation.

## Results

### Clinical History

Patient II.1 started autonomous walking at normal age (13 months) with abnormal plantar flexion of foot (equine’s foot, walking on his toes). He was followed since the age of 2 years for gait abnormalities. Neurological examinations described a spastic gait with hyperextension of both knees, swaying hips, and plantar flexion of the feet. He was diagnosed with Strümpell-Lorrain disease (HSP 56) at 3 years of age. To improve motor function, several surgical muscle extensions and botulinum toxin injections were performed. At baseline (age 25), the patient can walk autonomously but frequently uses a wheelchair. Cerebral and spinal magnetic resonance imaging performed at regular intervals during adolescence was within normal limits. Patient II.1 smokes cannabis on a regular basis for the relief of spasticity-related pain. He reported no subjective visual symptoms at any point in time. Patients I.1-2 (parents) and siblings II.2 and II.3 were neurologically asymptomatic. Patient II.3 had reduced visual acuity since approximately 15 years of age. He also reports photophobia starting in his twenties.

### *CYP2U1* Variants

Whole exome sequencing with bioinformatics analysis of a panel of HSP genes revealed NM_183075.2: c.452C>T; p. Pro151Leu, classified as likely pathogenic (according to American College of Medical Genetics and Genomics criteria), and NM_183075.2: c.943C>T; p. Gln315Ter, classified as pathogenic according to American College of Medical Genetics and Genomics criteria. Both variants were validated by Sanger sequencing; patients II.1 and II.3 were compound heterozygotes for both variants. Familial segregation confirmed that each parent (I.1 and I.2) is a heterozygous carrier of each variant. Patient II.2 is a carrier of the c.452C>T; p. Pro151Leu variant.

### Ophthalmic Examinations

Slit lamp biomicroscopy revealed normal anterior segments, intraocular pressures were within the normal range, and optic nerve head morphology was normal in all patients examined. Patients I.1, I.2, and II.2 were subjectively asymptomatic, performed functionally within normal parameters, and retinal imaging demonstrated no abnormalities in their phenotype. The retinal periphery was unremarkable in all our patients.

#### Patient II.1

##### Functional Testing

Best corrected visual acuity at the time of imaging was 0.12 logarithm of the minimum angle of resolution with the right eye and 0.14 logarithm of the minimum angle of resolution with the left (Snellen equivalent of 0.8 with either eye). Electrophysiological testing was attempted but was not feasible because of photophobia. Goldmann kinetic visual field testing revealed normal visual fields in both eyes. macular integrity assessment system microperimetry showed a mean retinal sensitivity of 25.6 decibels (dB) (median 25.0 dB, standard deviation [SD] 2.3 dB) with the right eye and 24.0 dB (median 25.0 dB, SD 3.3 dB) with the left eye. Ellipsoid zone breaks were too small for calculating mean sensitivity over retinal areas within the EZ lesions. Fixation stability (bivariate contour ellipse area) was 2.2°^2^ with the right eye and 6.5°^2^ with the left eye.

##### Multimodal Retinal Imaging

**Figure 2:**
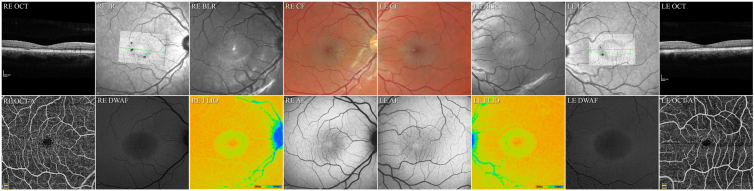
Multimodal retinal imaging of patient II.1. RE indicates the right eye, LE the left eye, color fundus (CF) images demonstrate a loss of retinal transparency in both eyes. Blue light reflectance (BLR) images show an increased scatter within the same area as the loss of retinal transparency. (In the RE, the bright round hyperreflectivity at the fovea and the faint round hyperreflectivity temporal inferior to the fovea are artifacts due to internal reflections within the optics of the recording device, the arcuate hyperreflectivity along the arcades in both eyes are retinal surface reflections). Infrared (IR) images with an overlay of the “*en face*” image of the EZ and a green line marking the position of the corresponding sample OCT B-scan traversing the foveal center. En face images of the EZ show in both eyes an attenuation of the signal within the parafovea with an outline coinciding to those seen in BLR, DWAF and FLIO images. In the RE multiple focal discontinuities (breaks) in the EZ are apparent within this area, temporally and inferiorly. In the LE the EZ breaks are located nasal to the fovea, within the papillomacular area. Within the OCT B-scan of the RE, a focal discontinuity in the photoreceptor outer segment line and the ellipsoide zone are apparent with a thinning/disorganization of the ONL as well as low reflective spaces within the inner retina. The OCT of the LE shows low reflective spaces within the inner retina and a slight attenuation of the in the photoreceptor outer segment line. The hyperreflectivity within the ONL in the nasal retinal is most likely due to an improved visibility of Henle’s layer due to a slight tilt of the scan. The OCT angiographic (OCTA) images show in the right eye a slightly enlarged, in the left eye a normal size FAZ with bordering dilated capillaries and increased intercapillary gaps. Fundus autofluorescence (AF) images show a loss of the normally present central hypo-AF peak and increased AF on the temporal side in both eyes whereas DWAF imaging demonstrates a generalized low macular pigment (MP) content within a ring of increased LP at approximately 6 degrees eccentricity. Fluorescence lifetime imaging ophthalmoscopy (color range: 200ps in red−to 500ps in blue) imaging shows a small central area with short fluorescence lifetimes surrounded by a clearly demarcated ring of prolonged lifetimes. The peripheral retina features a normal fluorescence lifetime distribution. DWAF = dual wavelength autofluorescence; EZ = ellipsoid zone; FLIO = fluorescence lifetime imaging ophthalmoscopy; LP = lipofuscin; ONL = outer nuclear layer.

Color fundus imaging revealed a loss of retinal transparency and BLR imaging a hyperreflectivity within the parafovea in both eyes (Fig 2). Color fundus and BLR imaging modalities were confounded by patchy retinal surface reflectivity. OCT imaging showed low reflective spaces in the inner retina within the parafovea, just internal to the outer nuclear layer (ONL) and internal to the inner nuclear layer. Small patches of low reflective spaces were also present in the outer retina (EZ breaks), at the edge of the parafovea, temporal and inferior to the foveal center in the right eye, and nasal to the fovea in the left eye. Blue (488 nm) AF imaging showed a loss of the central peak of macular pigment (MP) at the fovea; DWAF imaging revealed a depletion of MP within the parafovea with a ring of increased MP density at approximately 5.5° radial distance from the foveal center. Fluorescence Lifetime Imaging Ophthalmoscopy imaging showed short fluorescence lifetimes at the foveal center (central 1mm ETDRS subfield) with values of 250 to 264 ps (age-corrected reference value: 135 ± 21 ps). In the inner (3 mm) ETDRS ring, the values were prolonged to 278 to 284 ps (reference: 217 ± 23 ps), whereas, in the outer (6 mm) ETDRS ring and the peripheral retina, the values were normal with 258 to 260 ps (reference: 248 ± 16 ps). The ring of prolonged fluorescence lifetimes corresponded to a central area of changes in the infrared image and to a zone of irregular or missing photoreceptors in the OCT image. OCT angiography imaging revealed enlarged intercapillary spaces around the foveal avascular zone (FAZ) in both eyes. Foveal avascular zone size was within normal parameters.

#### Patient II.3

##### Functional Testing

**Figure 3:**
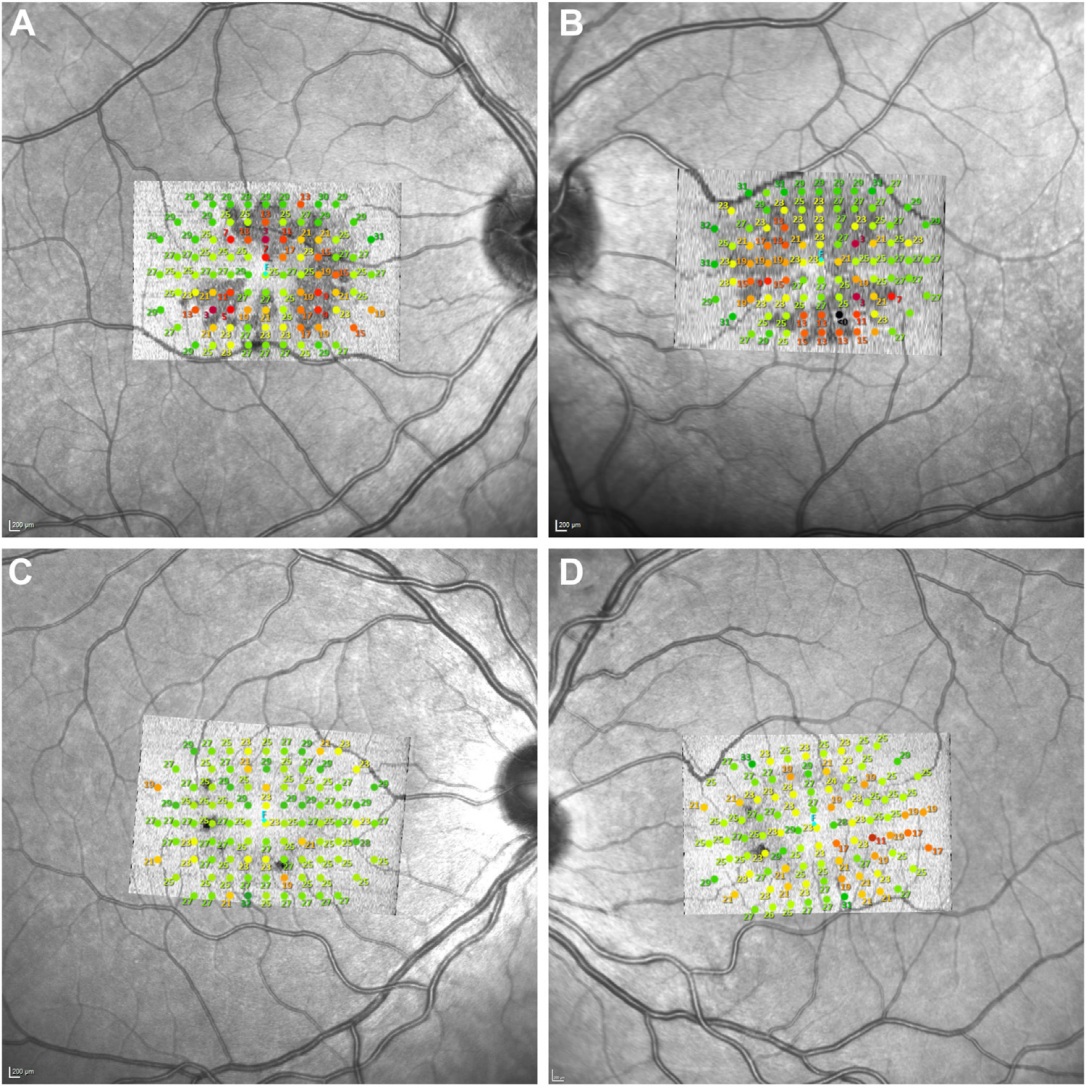
MAIA microperimetry and en face OCT images of the EZ. (A, B) The MAIA microperimetry measurements of the right and left eyes of patient II. 3., (C, D) Right and left eyes of patient II. 1. The background images are Heidelberg Spectralis infrared images, with aligned overlays of the en face OCT image of the EZ. The stimulus used was Goldmann III at a 1-degree spacing within the central area. **F**, Preferred locus of fixation. EZ = ellipsoid zone; MAIA = macular integrity assessment system.

Best corrected visual acuity at the time of imaging was logarithm of the minimum angle of resolution 0.1 with each eye (Snellen equivalent 0.8). International Society for Clinical Electrophysiology of Vision standard full-field ERGs, electrooculograms, and Goldmann kinetic visual fields were within normal limits. Microperimetry showed a mean retinal sensitivity within EZ breaks of 15.3 dB (median 17.0 dB, SD 6.9 dB) in the right eye and 16.2 dB (median 16.0 dB, SD 6.9 dB) in the left eye, and outside the EZ breaks of 25.7 dB (median 27.0 dB, SD 3.6 dB) with the right eye and 25.9 dB (median 27.0 dB, SD 3.2 dB) with the left eye (Fig 3). Fixation stability (bivariate contour ellipse area) was 0.1°^2^ with the right eye and 0.2°^2^ with the left eye.

##### Multimodal Retinal Imaging

**Figure 4:**
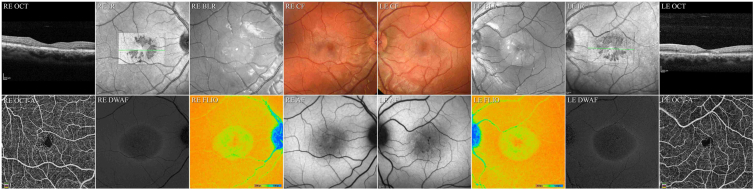
Multimodal retinal imaging of patient II.3. RE indicates the right eye, LE the left eye, color fundus (CF) images demonstrate a loss of retinal transparency in both eyes and small brown vessel-adjacent pigment foci in the left eye. Blue light reflectance (BLR) images show an increased scatter within the same area as the loss of retinal transparency. (In the RE, the small bright round hyperreflectivity and larger circular hyperreflectivity at the fovea are artifacts due to internal reflections within the optics of the recording device, the hyperreflectivity along the vessel arcades in both eyes are retinal surface reflections). Infrared (IR) images with an overlay of the “*en face*” image of the EZ and a green line marking the position of the corresponding sample OCT B-scan traversing the foveal center. En face images of the EZ show in both eyes a radial or petaloid arrangement of breaks in the EZ and a thinning of the ONL. Notable is the relative sparing on the temporal side, especially in the right eye. Small low reflective spaces are apparent within the inner retina. The OCT angiographic (OCTA) images show in both eyes an enlarged, deformed FAZ with bordering dilated capillaries and increased intercapillary gaps. Fundus autofluorescence (AF) images show a loss of the normally present central hypo-AF peak in both eyes. Small focal hypo-AF spots are apparent in both eyes some of these in the LE collocate with the brown focal vessel-adjacent pigment apparent in CF images. Dual wavelength autofluorescence (DWAF) imaging demonstrates a generalized low macular pigment (MP) content within a ring of increased LP at approximately 7 degrees eccentricity. Fluorescence lifetime imaging ophthalmoscopy (FLIO, color range: 200ps in red - to 500ps in blue) shows a slight central area with short fluorescence lifetimes surrounded by a clearly demarcated ring of prolonged lifetimes. This corresponds to the area of photoreceptor atrophy in the OCT image. The ring of prolonged fluorescence lifetimes features an external border with a small ring of short fluorescence lifetimes, fading out toward the retinal periphery, which features a normal fluorescence lifetime distribution. EZ = ellipsoid zone; FAZ = foveal avascular zone; LP = lipofuscin; ONL = outer nuclear layer.

**Figure 5:**
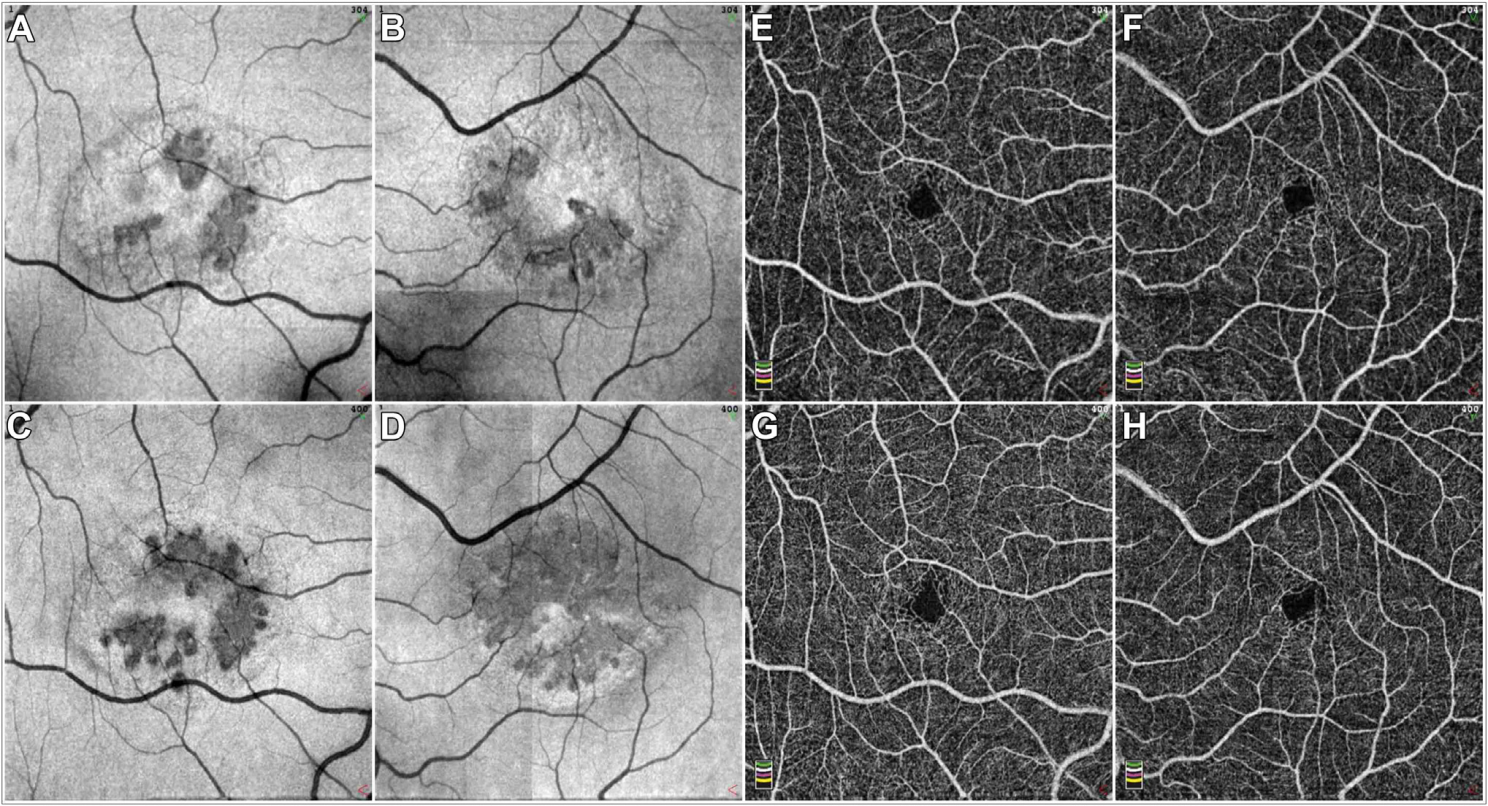
Progression patterns of neurodegenerative and vascular changes in patient II.3. **A–D**, en face OCT images of the EZ, (**A, C**) right eye, (**B, D**) left eye, (**E–H**) OCTA images, (**E, G**) right eye, (**F, H**) left eye, top row (**A, B, E, F**) 3 years before baseline, bottom row (**C, D, G, H**) at baseline. The EZ break apparent 3 years before baseline imaging show a radial distribution with no apparent predominance of the temporal area as in typical MacTel. At baseline the focal EZ loss appears to have progressed locally by an enlargement of the previously present foci as well as by the appearance of new foci, notably superior to the fovea in the left eye. OCTA images show an enlargement of the FAZ over time, with an increase in intercapillary gaps and in the left eye a radial arrangement of vessels around a focal brown pigment apparent in the CF image and as a focal hypo-AF in the AF image. This feature is characteristic of MacTel. AF = autofluorescence; CF = color fundus; EZ = ellipsoid zone; MacTel = macular telangiectasia type 2; OCTA = OCT angiography.

**Figure 6:**
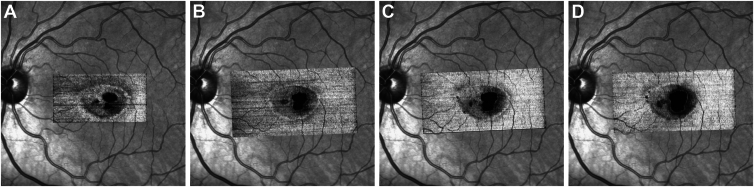
Progression pattern of EZ loss in a typical MacTel eye. In MacTel, EZ loss starts typically temporal to the foveal center, with time increases in all directions, a second locus may appear nasal to the foveal center. Visual acuity typically remains preserved until the EZ loss involves most of the fovea. The time interval between images A to D was 1 year. EZ = ellipsoid zone; MacTel = macular telangiectasia type 2.

Color fundus imaging revealed a bilateral loss of retinal transparency within the parafovea (Fig 4). In the left eye, small foci of perivascular pigmentation in the deeper layers of the retina were apparent. Blue light reflectance imaging showed a hyperreflectivity within the same region as the loss of retinal transparency. Color fundus and BLR imaging modalities were confounded by retinal surface reflectivity. OCT imaging showed within the parafovea an extensive outer retinal (photoreceptor) degeneration with loss of the interdigitation zone and EZ, and thinning or loss of the ONL, topographically in a petaloid pattern centered on the fovea, with foveal sparing in both eyes. The temporal parafovea appeared less affected in both eyes. Within the area of outer retinal atrophy, an inner retinal disorganization and granular hyperreflectivity were apparent. Low reflective intraretinal spaces were apparent just external to the retinal nerve fiber layer and to the outer plexiform layer. Corresponding to pigment seen in CF images and to hypo-AF, patches of increased scatter (hyperreflectivity) were detectable in the mid-outer retina, extending toward the retinal pigment epithelium. AF imaging showed an irregular depletion of central MP in both eyes and small hypo-AF foci associated with minor blood vessels, 2 in the right eye, 4 in the left eye. In the left eye, 1 hypo-AF focus appeared to join 2 smaller vessels (Fig 2, LE AF488nm). DWAF imaging revealed a depletion of MP within the parafovea with a ring of increased MP density at approximately 6° radial distance from the foveal center. Fluorescence Lifetime Imaging Ophthalmoscopy imaging showed short fluorescence lifetimes in the foveal center (1 mm) with values of 250 to 260 ps (age-corrected reference value: 135 ± 21 ps). In the inner ETDRS ring, the values were prolonged to 281 to 288 ps (reference: 217 ± 23 ps), whereas, in the 6 mm ETDRS ring and the peripheral retina, the values were normal at 249 to 253 ps (reference: 248 ± 16 ps). The outer border of the ring of prolonged fluorescence lifetimes corresponded approximately to a central area of hypo-AF in the autofluorescence intensity image, to a zone of irregular or missing photoreceptors in the OCT image, and featured a speckled pattern of irregularly prolonged lifetime areas. Outside this ring of prolonged lifetimes, an attenuated band of short lifetimes was observed. In OCTA images, an irregular, enlarged FAZ was apparent with surrounding wider intercapillary spaces in both eyes. In the left eye, capillaries demonstrated a radial pattern converging on a focal pigmented lesion temporal inferior to the foveal center. Historical OCT/OCTA images were available for patient II.3, from 3 years before baseline (Fig 5). EZ break area increased over this timespan by 95% in the right eye, and 77% in the left eye. The pattern appeared radial/petaloid with a sparing of the fovea and a relative sparing of the temporal part of the parafovea. Historical IR images and B-scans from 2013 demonstrate that the EZ breaks as seen in Figure 4A and B were already present, although the scan parameters did not permit break area measurements with reasonable accuracy. For comparison, a typical progression of EZ loss in a MacTel eye is illustrated in Figure 6.

## Discussion

Visual impairment is a frequent complication of HSPs,^15^ optic atrophy being the most frequently described, and it can also be an isolated finding.^29^ Early cataract, retinitis pigmentosa, and nystagmus have also been reported.^30^ An isolated maculopathy has been described in a few cases.^5^ Leonardi et al^10^ reported bilateral macular atrophy with retinal pigment epithelium changes, a mild temporal paleness of the optic nerve, and severely reduced BCVA in 3 patients with a homozygous c.1168C>T (p.R390∗) variant in *SPG56/CYP2U1*. Retinal anomalies were noted before the development of motor defects. Iodice et al^7^ reported hemorrhagic macular lesions preceding a “pigmentary maculopathy” in a 34-year-old female with c.1288?1G>A; and c.1545_1546delTTAC in CYP2U1; however, the phenotype was not described. Legrand et al^14^ observed in 3 patients with homozygote variants in *CYP2U1* a maculopathy, reduced central retinal thickness and outer retinal atrophy, retinal pigment epithelium atrophy (1 patient), and a hemorrhagic choroidal neovascular membrane (1 patient). The latter patient had the same homozygote *CYP2U1* c.1168C>T (p.R390∗) variant as the family described by Leonardi.^10^

Our fundoscopic and OCT findings are compatible with previous reports, our cases may represent a milder form and/or an earlier stage of the retinal phenotype. It may be that the retinal phenotype only manifests in bi-allelic variants in CYP2U1. The constellation of retinal anomalies of both affected patients, however, show striking similarities to the phenotype of MacTel.^17^

The MacTel phenotype includes characteristic neurodegenerative and vascular signs. A parafoveal loss of retinal transparency and a corresponding BLR hyperreflectivity are early signs of MacTel.^31^, ^32^, ^33^ Both affected patients demonstrated these, patient II.1 with similar parameters as in MacTel, whereas, in II.3 the abnormal area was 30% larger.^34^

Macular telangiectasia type 2 is characterized by a progressive pattern of MP redistribution, starting as a wedge-shaped depletion in the temporal parafovea, a subsequent loss of the central peak, and eventually a generalized depletion of MP within a ring of increased MP density at the edge of the parafovea. With the exception of Sjögren-Larsson syndrome,^35^ this pattern appears to be specific to MacTel. Both affected patients demonstrated the late stage of this pattern. As in MacTel, abnormal areas seen in DWAF and BLR images showed a close correspondence.^31^ Fluorescence Lifetime Imaging Ophthalmoscopy values at the fovea showed a depletion rather than an absence of short lifetime fluorophores, which could indicate residual MP and/or remaining central photoreceptors.^36^ Fluorescence Lifetime Imaging Ophthalmoscopy imaging showed a radially symmetrical concentric ring of prolonged lifetime values that collocated with OCT anomalies but were better visible, also in some areas with apparently normal photoreceptors. Interestingly, there was an increased density of shorter fluorescence lifetimes at the outer border of the ring, which is normally not the case at that eccentricity.^37^^,^^38^ The origin of this remains to be investigated.

A progressive parafoveal loss of the outer retina is also characteristic of MacTel. In patient II.1, bilateral focal EZ breaks were present: temporal/inferior in the right eye, nasal to the fovea in the left eye. Patient II.3 demonstrated a more extensive EZ loss restricted to the parafovea (as in MacTel), however predominantly on the nasal side, in a radial pattern and sparing the foveal center and the temporal parafovea, even more clearly in historical images recorded 3 years before baseline. These patterns are somewhat different from those seen in MacTel, which typically starts temporal to the foveal center progressing locally in all directions.^34^

Retinal sensitivity loss over EZ break areas was similar to that seen in MacTel, although of lower amplitude, that is, the scotomata were less “deep” than in MacTel.

Later stages of MacTel are characterized by perivascular brown pigment plaques. In the left eye of patient II.3, small pigment foci were detectable surrounding small vessels, similar to early-mid stage MacTel.^39^

MacTel is also associated with vascular signs. In patient II.3, OCTA showed a progressive enlargement and deformation of the FAZ, enlarged intercapillary spaces around the FAZ, and in the left eye, the vessel configuration indicated a focal lateral contraction of the retina,^40^ as frequently seen in MacTel. In patient II.1, the right eye demonstrated similar vascular changes, whereas the left eye appeared normal. Other vascular anomalies common in MacTel were not detected. Overall, neurodegenerative signs appeared to dominate the phenotype of both affected patients, more than what is typical for MacTel, although similar cases have also been reported in MacTel.^41^ Neovascularization - an optional late complication of MacTel - also reported in CYP2U1 mutations,^7^^,^^14^ was not evident in our patients.

The etiology and pathogenesis of MacTel are not fully understood. MacTel has a genetic component evidenced by extended families having multiple affected family members, however, determining a disease mechanism has been difficult because of the highly heterogeneous genetic architecture, and variable penetrance and expressivity of the disease.^19^^,^^20^^,^^21^^,^^42^ Recent studies have linked MacTel to low circulating levels of serine and glycine, which drive a concomitant increase in a neurotoxic lipid species, deoxy sphingolipids (deoxySL).^22^^,^^43^^,^^44^ The identification of a deoxySL-linked MacTel mechanism was facilitated by the discovery that rare mutations in the serine palmitoyl transferase (SPT) genes SPTLC1 and SPTLC2 directly elevate levels of deoxySL and are causative for a rare peripheral neuropathy, hereditary sensory and autonomic neuropathy type 1^45^^,^^46^ are also causative for MacTel.^43^^,^^47^ A subsequent whole exome sequencing screen identified functional variants in the gene encoding the rate-limiting serine biosynthetic enzyme, phosphoglycerate dehydrogenase which causes a reduction of serine synthesis and elevation of deoxySL synthesis.^42^ These variants are rare in MacTel patients, collectively accounting for less than 4% of the disease load; however, their identification was essential to uncovering a central disease mechanism linking MacTel to decreased serine to elevated deoxySL levels. It is apparent that serine levels and deoxySLs do not explain the entire functional disease mechanism, and the identification of additional causative mutations is critical to understanding contributing factors to the disease. Despite extensive whole exome sequencing studies, causal genes and variants leading to MacTel disease have remained elusive. Similar to the discovery of SPT mutations, here, we present findings that identify HSP and a phenotype compatible with MacTel in patients with *CYP2U1* variants, indicating potentially new disease mechanisms.

The *CYP2U1* gene encodes a member of the cytochrome P450 family, enzymes known to play key roles in tissue-specific conversion of natural substrates into locally active hormones, vitamins, and signaling molecules. Pujol et al^4^ found in both humans and rodents that a loss of *CYP2U1* impacts mitochondrial activity essential for various processes, such as lipid import or apoptosis, whose dysfunction is closely associated with diseases.^48^

The potential role of *CYP2U1* in mitochondrial function and the onset of MacTel is consistent with recent findings by Birtel et al^49^ who reported 2 cases of chronic progressive external ophthalmoplegia associated with sporadic single mtDNA deletions (m.11037_14597del), demonstrating some phenotypic characteristics associated with MacTel. The authors hypothesized that MacTel may involve mitochondrial dysfunction^50^ and noted that the susceptibility loci identified by Scerri et al^22^ include genes encoding mitochondrial enzymes and that low serine levels and increased levels of deoxy sphingolipids have previously been linked to disrupted mitochondrial function.^43^^,^^51^^,^^52^

In conclusion, we demonstrate that CYP2U1 variants are associated with a retinal phenotype very similar to that otherwise specific to MacTel, suggestive of possible links in the etiology and/or pathogenesis of these diseases. Retinal screening of patients suffering from neuromuscular diseases may provide clues to an early diagnosis and the pathogenetic pathways involved. CYP2U1 should be included in panels of genes involved in macular dystrophies.

## References

[1] Fink J.K. (2013). Hereditary spastic paraplegia: clinico-pathologic features and emerging molecular mechanisms. Acta Neuropathol.

[2] Tesson C., Koht J., Stevanin G. (2015). Delving into the complexity of hereditary spastic paraplegias: how unexpected phenotypes and inheritance modes are revolutionizing their nosology. Hum Genet.

[3] Chuang S.S., Helvig C., Taimi M. (2004). *CYP2U1*, a novel human thymus- and brain-specific cytochrome P450, catalyzes omega- and (omega-1)-hydroxylation of fatty acids. J Biol Chem.

[4] Pujol C., Legrand A., Parodi L. (2021). Implication of folate deficiency in *CYP2U1* loss of function. J Exp Med.

[5] Tesson C., Nawara M., Salih M.A. (2012). Alteration of fatty-acid-metabolizing enzymes affects mitochondrial form and function in hereditary spastic paraplegia. Am J Hum Genet.

[6] Citterio A., Arnoldi A., Panzeri E. (2014). Mutations in *CYP2U1, DDHD2* and *GBA2* genes are rare causes of complicated forms of hereditary spastic paraparesis. J Neurol.

[7] Iodice A., Panteghini C., Spagnoli C. (2017). Long-term follow-up in spastic paraplegia due to *SPG56/CYP2U1*: age-dependency rather than genetic variability?. J Neurol.

[8] Kariminejad A., Schöls L., Schüle R. (2016). *CYP2U1* mutations in two Iranian patients with activity induced dystonia, motor regression and spastic paraplegia. Eur J Paediatr Neurol.

[9] Kumar K.R., Wali G.M., Kamate M. (2016). Defining the genetic basis of early onset hereditary spastic paraplegia using whole genome sequencing. Neurogenetics.

[10] Leonardi L., Ziccardi L., Marcotulli C. (2016). Pigmentary degenerative maculopathy as prominent phenotype in an Italian *SPG56/CYP2U1* family. J Neurol.

[11] Masciullo M., Tessa A., Perazza S. (2016). Hereditary spastic paraplegia: novel mutations and expansion of the phenotype variability in SPG56. Eur J Paediatr Neurol.

[12] Durand C.M., Dhers L., Tesson C. (2018). *CYP2U1* activity is altered by missense mutations in hereditary spastic paraplegia 56. Hum Mutat.

[13] Minase G., Miyatake S., Nabatame S. (2017). An atypical case of *SPG56/CYP2U1*-related spastic paraplegia presenting with delayed myelination. J Hum Genet.

[14] Legrand A., Pujol C., Durand C.M. (2021). Pseudoxanthoma elasticum overlaps hereditary spastic paraplegia type 56. J Intern Med.

[15] Lo Giudice T., Lombardi F., Santorelli F.M. (2014). Hereditary spastic paraplegia: clinical-genetic characteristics and evolving molecular mechanisms. Exp Neurol.

[16] Zenteno J.C., Arce-Gonzalez R., Matsui R. (2023). Clinical-genetic findings in a group of subjects with macular dystrophies due to mutations in rare inherited retinopathy genes. Graefes Arch Clin Exp Ophthalmol.

[17] Charbel Issa P., Gillies M.C., Chew E.Y. (2013). Macular telangiectasia type 2. Prog Retin Eye Res.

[18] Gass J.D., Blodi B.A. (1993). Idiopathic juxtafoveolar retinal telangiectasis. Update of classification and follow-up study. Ophthalmology.

[19] Ronquillo C.C., Wegner K., Calvo C.M., Bernstein P.S. (2018). Genetic penetrance of macular telangiectasia type 2. JAMA Ophthalmol.

[20] Parmalee N.L., Schubert C., Figueroa M. (2012). Identification of a potential susceptibility locus for macular telangiectasia type 2. PLOS ONE.

[21] Parmalee N.L., Schubert C., Merriam J.E. (2010). Analysis of candidate genes for macular telangiectasia type 2. Mol Vis.

[22] Scerri T.S., Quaglieri A., Cai C. (2017). Genome-wide analyses identify common variants associated with macular telangiectasia type 2. Nat Genet.

[23] Richards S., Aziz N., Bale S. (2015). Standards and guidelines for the interpretation of sequence variants: a joint consensus recommendation of the American College of Medical Genetics and Genomics and the Association for Molecular Pathology. Genet Med.

[24] Dysli C., Wolf S., Berezin M.Y. (2017). Fluorescence lifetime imaging ophthalmoscopy. Prog Retin Eye Res.

[25] Dysli C., Quellec G., Abegg M. (2014). Quantitative analysis of fluorescence lifetime measurements of the macula using the fluorescence lifetime imaging ophthalmoscope in healthy subjects. Invest Ophthalmol Vis Sci.

[26] Chew E.Y., Clemons T.E., Jaffe G.J. (2019). Effect of ciliary neurotrophic factor on retinal neurodegeneration in patients with macular telangiectasia type 2: a randomized clinical trial. Ophthalmology.

[27] Sallo F.B., Peto T., Egan C. (2012). “En face” OCT imaging of the IS/OS junction line in type 2 idiopathic macular telangiectasia. Invest Ophthalmol Vis Sci.

[28] Crossland M.D., Dunbar H.M., Rubin G.S. (2009). Fixation stability measurement using the MP1 microperimeter. Retina.

[29] Klebe S., Depienne C., Gerber S. (2012). Spastic paraplegia gene 7 in patients with spasticity and/or optic neuropathy. Brain.

[30] de Freitas J.L., Rezende Filho F.M., Sallum J.M.F. (2020). Ophthalmological changes in hereditary spastic paraplegia and other genetic diseases with spastic paraplegia. J Neurol Sci.

[31] Sallo F.B., Leung I., Zeimer M. (2018). Abnormal retinal reflectivity to short-wavelength light in type 2 idiopathic macular telangiectasia. Retina.

[32] Charbel Issa P., Berendschot T.T., Staurenghi G. (2008). Confocal blue reflectance imaging in type 2 idiopathic macular telangiectasia. Invest Ophthalmol Vis Sci.

[33] Heeren T.F.C., Chew E.Y., Clemons T. (2020). Macular telangiectasia type 2: visual acuity, disease end stage, and the MacTel Area: MacTel Project Report Number 8. Ophthalmology.

[34] Pauleikhoff D., Bonelli R., Dubis A.M. (2019). Progression characteristics of ellipsoid zone loss in macular telangiectasia type 2. Acta Ophthalmol.

[35] van der Veen R.L., Fuijkschot J., Willemsen M.A. (2010). Patients with Sjögren-Larsson syndrome lack macular pigment. Ophthalmology.

[36] Sauer L., Andersen K.M., Li B. (2018). Fluorescence lifetime imaging ophthalmoscopy (FLIO) of macular pigment. Invest Ophthalmol Vis Sci.

[37] Sauer L., Gensure R.H., Hammer M., Bernstein P.S. (2018). Fluorescence lifetime imaging ophthalmoscopy: a novel way to assess macular telangiectasia type 2. Ophthalmol Retina.

[38] Dysli C., Berger L., Wolf S., Zinkernagel M.S. (2017). Fundus autofluorescence lifetimes and central serous chorioretinopathy. Retina.

[39] Leung I., Sallo F.B., Bonelli R. (2018). Characteristics of pigmented lesions in type 2 idiopathic macular telangiectasia. Retina.

[40] Sallo F.B., Peto T., Egan C. (2012). The IS/OS junction layer in the natural history of type 2 idiopathic macular telangiectasia. Invest Ophthalmol Vis Sci.

[41] Gillies M.C., Mehta H., Bird A.C. (2015). Macular telangiectasia type 2 without clinically detectable vasculopathy. JAMA Ophthalmol.

[42] Eade K., Gantner M.L., Hostyk J.A. (2021). Serine biosynthesis defect due to haploinsufficiency of PHGDH causes retinal disease. Nat Metab.

[43] Gantner M.L., Eade K., Wallace M. (2019). Serine and lipid metabolism in macular disease and peripheral neuropathy. N Engl J Med.

[44] Bonelli R., Ansell B.R.E., Lotta L. (2021). Genetic disruption of serine biosynthesis is a key driver of macular telangiectasia type 2 aetiology and progression. Genome Med.

[45] Penno A., Reilly M.M., Houlden H. (2010). Hereditary sensory neuropathy type 1 is caused by the accumulation of two neurotoxic sphingolipids. J Biol Chem.

[46] Rotthier A., Auer-Grumbach M., Janssens K. (2010). Mutations in the SPTLC2 subunit of serine palmitoyltransferase cause hereditary sensory and autonomic neuropathy type I. Am J Hum Genet.

[47] Duan J., Merrill A.H. (2015). 1-Deoxysphingolipids encountered exogenously and made de novo: dangerous mysteries inside an enigma. J Biol Chem.

[48] Scorrano L., De Matteis M.A., Emr S. (2019). Coming together to define membrane contact sites. Nat Commun.

[49] Birtel J., von Landenberg C., Gliem M. (2022). Mitochondrial retinopathy. Ophthalmol Retina.

[50] Zucker C.L., Bernstein P.S., Schalek R.L. (2020). A connectomics approach to understanding a retinal disease. Proc Natl Acad Sci U S A.

[51] Alecu I., Tedeschi A., Behler N. (2017). Localization of 1-deoxysphingolipids to mitochondria induces mitochondrial dysfunction. J Lipid Res.

[52] Zhang T., Gillies M.C., Madigan M.C. (2018). Disruption of de novo serine synthesis in Müller cells induced mitochondrial dysfunction and aggravated oxidative damage. Mol Neurobiol.

